# Pricing Carbon and Adjusting Capital to Fend Off Climate Catastrophes

**DOI:** 10.1007/s10640-018-0231-2

**Published:** 2018-02-26

**Authors:** Frederick van der Ploeg, Aart de Zeeuw

**Affiliations:** 1 Department of Economics, University of Oxford, Oxford, OX1 3UQ UK; 2 Department of Economics, Tilburg University, P.O. Box 90153, 5000 LE Tilburg, The Netherlands; 3 Vrije Universiteit, Amsterdam, The Netherlands; 4 Beijer Institute of Ecological Economics, The Royal Swedish Academy of Sciences, Stockholm, Sweden

**Keywords:** Climate tipping point, Risk, Social cost of carbon, Precautionary capital, Economic growth, D81, H20, O40, Q31, Q38

## Abstract

The optimal reaction to a potential productivity shock as a consequence of climate tipping is to substantially tax carbon in order to curb the risk of tipping, but to adjust capital as well in order to smooth consumption when tipping occurs. We also allow for conventional marginal climate damages and decompose the optimal carbon tax in two catastrophe components and the conventional component. We distinguish constant and increasing marginal hazards. Moreover, the productivity catastrophe is compared with recoverable catastrophes and with a shock to the climate sensitivity. Finally, we allow for investments in adaptation capital as an alternative to counter the potential adverse effects of climate tipping. Quantitatively, the results are investigated with a calibrated model for the world economy.

## Introduction

The standard recipe in handling climate change is to call for a global tax on carbon that is equal to the present value of all future marginal damages arising from emitting one ton of carbon today (e.g., Nordhaus 2008; Stern 2007; Golosov et al. 2014). However, climatologists emphasise that the real threats of climate change are the non-marginal effects when the climate system shifts to a system with different dominant feedbacks and different equilibria (Lenton and Ciscar 2013). The point where such a shift occurs is called a tipping point (Biggs et al. 2012). The literature on the economics of climate change has started to incorporate tipping points in the analysis (e.g., Kopits et al. 2013; Pindyck 2013). For example, several authors have used the DICE model—a prominent integrated assessment model for climate change (Nordhaus 2008)—to study the effect of climate tipping points (Keller et al. 2004; Lemoine and Traeger 2014; Cai et al. 2015; Lontzek et al. 2015).

This paper focuses first on theory, and more specifically on a potential climate shock that would lead to an adverse productivity shock in a Ramsey growth model, with uncertainty about the time, impact and type of such a climate catastrophe. The productivity shocks may arise from flooding of cities, sudden increased occurrence of storms and droughts, abrupt desertification of agricultural land, or reversal of the Gulf Stream (e.g., Alley et al. 2003). We show that the economy needs to respond in two ways to such potential shocks. First, carbon needs to be taxed (or priced via a global market for carbon emission permits) to curb the risk of climate calamities as the hazard of a catastrophe increases in the stock of atmospheric carbon. Second, precautionary adjustment to the capital stock is needed to cope with the possibility of an abrupt jump in consumption at the tipping point. The second response follows from adjustments to the Keynes–Ramsey rule in the Ramsey growth model with tipping, which is the focus of a companion paper (van der Ploeg and de Zeeuw 2018). This paper focuses, in contrast, on the social cost of carbon or the optimal carbon tax.

In order to make our results comparable with early literature on integrated climate assessment, we also allow for *gradual* marginal climate damages (e.g., Tol 2002; Nordhaus 2008; Stern 2007; Golosov et al. 2014). We show that the optimal carbon tax consists of two catastrophe components and the usual component internalising marginal damages. The catastrophe components are the risk-averting part and, what we call, the raising-the-stakes part. The first component reduces the probability of tipping, whereas the second component internalises the effects on expected after-calamity welfare. We offer explicit expressions for the optimal tax on carbon and we show, in a calibrated Ramsey growth model for the world economy, how big the increase in the carbon tax is, as compared to the carbon tax that is only based on marginal damages.

In this paper, we consider other types of shocks caused by climate tipping as well. A sudden acceleration of carbon emissions, because of a reduction in cooling when ice sheets such as the Western Antarctic Ice Sheet melt away (e.g., Oppenheimer 1998),^1^ or a sudden release of methane buried in sea-beds and permafrost (e.g., Dutta et al. 2006), because of rising sea temperatures, are other tipping points. We view them as a sudden increase in the climate sensitivity, which is the temperature rise from doubling the carbon stock, and we derive the optimal response to a potential tipping point in the climate sensitivity. We also consider the implications of *recoverable* catastrophes in this paper, such as a sudden rise in the carbon stock or a sudden destruction of a part of the capital stock. Furthermore, we show the effect of an increasing marginal hazard rate (as a function of the stock of atmospheric carbon), which captures the increasing marginal probability of tipping. Finally, we allow for investments in adaptation capital, as an alternative way to counter potential adverse effects of climate tipping.

This paper emphasises the need for an appropriate carbon tax, besides adjustments in saving, in the face of potential tipping points driven by global warming in a standard Ramsey growth model. Clarke and Reed (1994), Tsur and Zemel (1996) and Gjerde et al. (1999) also analyse catastrophes with hazard rates, but they do not have Ramsey growth and thus no need for additional adjustments in capital. Gjerde et al. (1999) consider carbon cycle and temperature modules with gradual and catastrophic damages and provide simulation studies of the effects of pending catastrophes on temperature and the economy, but they do not present the optimal carbon tax or the need for adjustments in saving. Smulders et al. (2014) discuss the need for precautionary saving to deal with an impending disaster, but their analysis uses a constant hazard rate, whereas our analysis highlights the effects of endogenous hazard rates that depend on global warming (represented by the carbon stock). Engström and Gars (2016) study catastrophes within the model of Golosov et al. (2014), but the special assumptions needed to get tractable solutions of this model remove the incentive to have adjustment in saving to cope with a pending catastrophe. Our study focuses on the theory first, in a Ramsey growth model, to highlight the need for an additional tax on carbon as well as adjustments to capital, and then presents illustrative quantitative results in a simple calibrated model of the world economy.

Other studies have focused on catastrophes and extreme events but not in the context of general equilibrium models of Ramsey growth and climate change. Barro (2013) studies the optimal investment needed to curb the probability of an environmental disaster but abstracts from the precautionary adjustment of capital. Weitzman (2007) highlights the fundamental uncertainty at the upper end of the probability distribution of possible increases in temperature and corresponding damage, and shows that the policy consequences of a fat instead of a thin tail can be dramatic. Martin and Pindyck (2015) study the ‘strange’ implications for the cost–benefit analysis of a cascade of catastrophes within a partial equilibrium context. Pindyck and Wang (2013) use a general equilibrium framework to obtain a price of insurance against catastrophic risks.

Section 2 presents our model of growth with tipping points and only catastrophic damages. Section 3 shows that the optimal response requires precautionary capital adjustment and, if the hazard increases with atmospheric carbon, taxing carbon to curb the risk of tipping. Section 4 allows for gradual as well as catastrophic damages from global warming and decomposes the optimal carbon tax into the usual social cost of carbon (i.e., the present value of marginal gradual damages) and the extra carbon taxes that are needed to curb the risk of climate tipping. It also discusses regime shifts with a sudden increase in climate sensitivity, and recoverable catastrophes that destroy a part of the capital stock or that lead to a sudden release of atmospheric carbon. Finally, investments in adaptation capital are discussed. Section 5 concludes.

## Ramsey Growth with Climate Tipping: Only Catastrophic Damages

Consider a continuous-time Ramsey growth model with a constant population. Fossil fuel *E* is input into the production process, has constant marginal cost *d* > 0, and (given abundance of coal and shale gas) is in abundant supply. There is a carbon-free imperfect substitute for fossil fuel *R*, renewable energy, with constant cost *c* > 0. Capital *K*, fossil fuel and the renewable substitute are cooperative factors of production. Total factor productivity is *A* before the regime shift and drops to $(1 - \pi )A < A$ afterwards, where 0 < *π* < 1 is the size of the climate disaster. Utility is denoted by *U*, consumption by *C*, the production function by *A* times *F* before and $(1 - \pi )A$ times *F* after the regime shift, the depreciation rate of capital by *δ* > 0 and the pure rate of time preference by *ρ* > 0. Use of fossil fuels leads to emissions of carbon dioxide with the emission rate *ψ* > 0. The stock of atmospheric carbon *P* decays naturally at the rate *γ* > 0 (typically about 1/300).^2^ To focus on the policy implications of tipping points, we abstract from population growth, carbon capture and carbon sequestration, learning-by-doing in the renewable sector, and other forms of technical progress. The model is very similar to the model used in van der Ploeg and de Zeeuw (2016), but in that paper the basic model is extended to two regions in order to analyse cooperative and non-cooperative responses to climate tipping between the North and the South.

We assume that the magnitude of the potential drop in total factor productivity is known but that it is not known *when* the climate regime shift will take place. The hazard rate *h* gives the conditional probability of the tipping point *T*. Formally,

1 $$ h(t) = \mathop {\lim }\limits_{\Delta t \to 0} \frac{\Pr [T \in (t,t + \Delta t)|T \notin (0,t)]}{\Delta t}, $$

so *h*(*t*)Δ*t* is the probability that the regime shift takes place between *t* and *t* + Δ*t*, given that it has not occurred before *t*. A constant hazard rate *h* has the exponential density function $$ f(t) = he^{ - ht} $$ with mean 1/*h* and cumulative density function $$ F(t) = 1 - e^{ - ht} $$. The probability of “survival” is *e*^−*ht*^. If the hazard rate *h* is not constant, *ht* is replaced by $$ \int_{0}^{t} {h(s)ds} . $$ This implies that the cumulative probability of the catastrophe occurring at some time *t* in the interval [0, *T*] is given by $$ 1 - \exp \left( { - \int_{0}^{T} {h(s)ds} } \right). $$ A larger stock of carbon *P* increases the probability of climate change. Therefore, we assume that the hazard rate depends on the carbon stock: $$ h(t) = H\left( {P(t)} \right) $$ with $$ H^{\prime}(P) > 0 $$. It follows that with global warming, the expected duration before the regime shift occurs, i.e. $$ 1/H(P), $$ decreases over time. Hence, failing climate policy makes the potential shock to productivity more imminent.

The social planner maximises the expected value of social welfare

2 $$ \mathop {\hbox{max} }\limits_{C,E,R} \,{\text{E}}\left[ {\int\limits_{0}^{\infty } {e^{ - \rho t} U(C(t))dt} } \right] $$

subject to the accumulation of capital and greenhouse gases

3 $$ \begin{aligned} \dot{K}(t) & = \tilde{A}(t)F\left( {K(t),E(t),R(t)} \right) - dE(t) - cR(t) - C(t) - \delta K(t), \\ \dot{P}(t) & = \psi E(t) - \gamma P(t),\quad K(0) = K_{0} ,\quad P(0) = P_{0} , \\ \end{aligned} $$

with total factor productivity given by

4 $$ \tilde{A}(t) = A,\quad 0 \le t < T,\quad \tilde{A}(t) = (1 - \pi )A < A,\quad t \ge T,\quad 0 < \pi < 1, $$

where the tipping point *T* is driven by the hazard rate *H*(*P*).

We solve this problem by backward induction. After the climate regime shift, the problem is a standard Ramsey growth model with total factor productivity $(1 - \pi )A$, which yields the optimal consumption path $C^{A} = C^{A} (K,\pi )$ and the value function $V^{A} (K,\pi )$ (see “Appendix 1”). Before the climate regime shift, the problem becomes a stochastic optimal control problem. However, because we have modelled uncertainty by means of a hazard rate, it can be transformed into a deterministic one (Clarke and Reed 1994). This will become clear in the next section.

## The Optimal Response Before the Regime Shift

The before-tip problem is to choose the rate of consumption and energy use in order to

5 $$ \mathop {\hbox{max} }\limits_{C,E,R} \;{\text{E}}\left[ {\int\limits_{0}^{T} {e^{ - \rho t} U\left( {C(t)} \right)dt} + e^{ - rT} V^{A} \left( {K(T),\pi } \right)} \right] $$

subject to the capital and carbon stock dynamics (3). The question is how the prospect of a potential climate regime shift, and the fact that this prospect becomes more imminent as the stock of atmospheric carbon and temperature rise, affect the optimal growth path before the tip occurs. We denote before-tip values with superscript *B*. Since the stock of atmospheric carbon increases the hazard rate, *H*(*P*), the before-tip value function, $V^{B} (K,P)$, depends on *P*. The deterministic Hamilton–Jacobi-Bellman equation for the before-tip problem becomes (e.g., Polasky et al. 2011)

6 $$ \begin{aligned} \rho V^{B} (K,P) & = \mathop {\text{Max}}\limits_{C,E,R} \left[ {U(C) - H(P)\left\{ {V^{B} (K,P) - V^{A} (K)} \right\}} \right. \\ & \quad \left. +\, {V_{K}^{B} (K,P)\left\{ {AF(K,E,R) - dE - cR - C - \delta K} \right\} + V_{P}^{B} (K,P)(\psi E - \gamma P)} \right], \\ \end{aligned} $$

with the optimality conditions

7 $$ U^{\prime } (C^{B} ) = V_{K}^{B} (K,P),\quad AF_{E} (K,E^{B} ,R^{B} ) = d + \tau ,\quad AF_{R} (K,E^{B} ,R^{B} ) = c, $$

where the *social cost of carbon, τ,* is the marginal cost of carbon emissions expressed in final goods units^3^:

8 $$ \tau = \psi \frac{{ - V_{P}^{B} (K,P)}}{{V_{K}^{B} (K,P)}}. $$

The second term on the right-hand side of (6) captures the expected capitalised loss from a regime shift that occurs at some unknown future date. With a zero hazard rate, the standard Ramsey growth model results, with total factor productivity equal to *A*. We call this the *naive* solution, since the potential regime shift is effectively ignored.

In “Appendix 2” it is shown that the solution yields the differential equation for the price of carbon *τ*

9 $$ \dot{\tau } = \left[ {Y_{K}^{B} (K,\tau ) + \gamma + H(P) + \theta } \right]\tau - \frac{{\psi H^{\prime } (P)\left[ {V^{B} (K,P) - V^{A} (K)} \right]}}{{U^{\prime } (C^{B} )}}, $$

and the modified Keynes–Ramsey rule

10 $$ \dot{C}^{B} = \sigma \left[ {Y_{K}^{B} (K,\tau ) + \theta - \rho } \right]C^{B} ,\quad \theta \equiv H(P)\left[ {\frac{{U^{\prime } (C^{A} )}}{{U^{\prime } (C^{B} )}} - 1} \right], $$

where *σ* denotes the elasticity of intertemporal substitution (*EIS*) and *Y* the maximum output level net of input costs and capital depreciation, and where we define *θ* as the precautionary return.

Consumption growth is thus proportional to the marginal net product of capital *plus* the precautionary return *θ* and *minus* the pure rate of time preference *ρ*. Typically the precautionary return on capital adjustments *θ* is positive in this case, and consumption jumps down at the tipping point. However, it is also possible that *θ* is negative, so that consumption jumps up at the tipping point. This can happen in case of large delays of the impact of the catastrophe (see van der Ploeg and de Zeeuw 2018). Notice that in a doomsday scenario where all the value is destroyed, i.e. $V^{A} (K) = 0$, the hazard rate *H*(*P*) must just be added to the discount rate *ρ*, so that $\theta = - H(P) < 0.$ Before tipping, consumption is then always higher and capital accumulation is lower than in the naive outcome. However, with life after the shock, discounting increases as well, but *θ* will be positive when this is offset by the precautionary effect (Polasky et al. 2011).

### The Carbon Tax

The optimal carbon tax is given by Eq. (9). This carbon tax arises in the presence of a tipping point and is therefore fundamentally different from the standard carbon tax that is equal to the present value of the stream of future marginal damages. Moreover, this carbon tax only arises if the hazard of tipping is endogenous, i.e. if it can influence economic behaviour in order to reduce the hazard of tipping. With a constant hazard, i.e. $H^{{\prime }} (P) = 0,$ tipping is exogenous, so that *τ* = 0 is the solution of (9) and the social optimum only requires precautionary saving adjustments. With $H^{\prime } (P) > 0$, however, there is a positive carbon tax *τ* to encourage substitution away from the use of fossil fuel and decrease the risk of tipping.

Integration of (9) gives the optimal carbon tax in the presence of potential tipping as the present value of the expected loss in welfare from a future disaster at an unknown point in time:

11 $$ \begin{aligned} \tau (t) & = \psi \int_{t}^{\infty } {e^{{ - \int_{t}^{s} {r^{Y} (s')ds'} }} \frac{{H^{\prime } \left( {P(s)} \right)\left[ {V^{B} \left( {K(s),P(s)} \right) - V^{A} \left( {K(s)} \right)} \right]}}{{U^{\prime } \left( {C^{B} (s)} \right)}}ds} \\ & = \frac{{\psi \int_{t}^{\infty } {e^{{ - \int_{t}^{s} {r^{U} (s')ds'} }} H^{\prime } \left( {P(s)} \right)\left[ {V^{B} \left( {K(s),P(s)} \right) - V^{A} \left( {K(s)} \right)} \right]ds} }}{{U^{\prime } \left( {C^{B} (t)} \right)}}, \\ \end{aligned} $$

where $r^{Y} \equiv Y_{K}^{B} (K,\tau ) + \gamma + H(P) + \theta$ denotes the rate used to discount utility in final goods units and $r^{U} \equiv \rho + \gamma + H(P)$ the rate to discount utility units. The discount rate includes the rate of decay of atmospheric carbon *γ* and the hazard rate *H(P)* itself. This is the discounting effect of the hazard rate that would be the only effect if all value was destroyed at the tipping point, as was mentioned above. The rate used to discount final goods units includes the precautionary return on capital adjustments *θ* whereas the rate used to discount utility units does not. It is important to note from Eq. (11) that the carbon tax is large if the drop in welfare from a climate calamity is large and if the marginal hazard rate is large. This implies that a convex hazard rate, as a function of the stock of carbon, will push up the carbon tax more than a linear hazard rate, because the marginal hazard of tipping is increasing. We will show how large this effect is in the numerical illustration below.

It is interesting to investigate the interaction of the carbon tax with the precautionary saving adjustments. If the capital stock is pushed up, the demand for energy becomes larger, and this may require a larger carbon tax. We will investigate this issue in the numerical illustration below. The opposite may happen as well but, as we will show below, for reasonable parameter values precautionary saving is positive. Finally, it is important to get an idea how big the carbon tax in Eq. (11) is, as compared to the standard carbon tax that is equal to the present value of the stream of future marginal damages. Therefore, in Sect. 4 we will add this carbon tax and we will show, both in theory and in numbers, using a calibrated model of the world economy, how the total optimal carbon tax is composed.

Note that a higher elasticity of substitution *σ* corresponds to both a lower coefficient of relative intergenerational inequality aversion (*CRIA*) and a lower coefficient of relative risk aversion (*CRRA*), and thus has two effects. A higher elasticity of substitution *σ* induces a lower precautionary return *θ* and less precautionary capital accumulation. Effectively, society has a lower *CRIA* and is thus less willing to sacrifice consumption and accumulate precautionary capital to be prepared for the eventual shock. As a result of using less capital in production, there will be less use of fossil fuel and thus carbon emissions will be lower. This yields a lower carbon tax. Furthermore, a lower *CRRA* implies that society is less willing to avert risk of a climate catastrophe and this reduces the carbon tax *τ* too. The net effect on the stock of atmospheric carbon is ambiguous, since a lower *CRIA* induces less precautionary capital accumulation and thus less fossil fuel use and carbon emissions, whereas a lower *CRRA* reduces the optimal carbon tax so that carbon emissions are reduced less and the carbon stock is higher. We will investigate the net effect in the numerical illustration below.

### Simulations with Hazard Rates that Rise with Global Warming

To illustrate these results, we assume that the initial carbon stock is *P*_0_ = 826 GtC and the hazard rate is *H*(826) = 0.025 which increases to *H*(1652) = 0.067. So, as the stock of atmospheric carbon doubles and global warming increases by 3 °C (using a climate sensitivity of 3), the average time it takes for the tip to occur drops from 40 to 15 years. The within-year risks of a catastrophe are thus 2.5% to start off with and rise by 4.2% once temperature has risen by 3 °C. Suppose that at the end of the next half century the temperature is 3 °C higher and that the hazard rate rises linearly. Using the expression for the cumulative hazard in Sect. 2, below Eq. (1), we can show that the cumulative risk of a catastrophe occurring during this half century is 91%: $1 - \exp \left( { - \int_{0}^{50} {\left[ {0.025 + 0.00042s} \right]ds} } \right) = 0.907.$

We calibrate both a linear and a quadratic hazard function that satisfy the conditions *H*(826) = 0.025 and *H*(1652) = 0.067:

12 $$ H_{1} (P) = 0.025 + 5.04 \times 10^{ - 5} (P - 826),\quad \quad H_{2} (P) = 0.025 + 6.11 \times 10^{ - 8} (P - 826)^{2} . $$

**Fig. 1:**
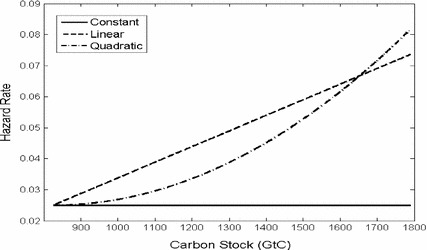
Different specifications for the hazard function

Both functions are depicted in Fig. 1.

The main difference is that the quadratic hazard rate is lower than the linear one at lower stocks of atmospheric carbon, but that it increases more sharply at higher stocks of atmospheric carbon. At higher stocks of atmospheric carbon, the quadratic hazard function leads to higher hazard rates than the linear one. For example, if the carbon stock quadruples to 3304 GtC (and global warming increases by an additional 6 °C), the hazard rate increases to 40% per annum with the quadratic and 15% with the linear hazard function.

Table 1 reports the steady states for different scenarios.^4^ With a constant hazard rate, the capital stock is pushed up to enable smoothing of consumption that jumps down in the event of a sudden drop in factor productivity, but fossil-fuel use and the stock of atmospheric carbon are pushed up too. However, if the risk of catastrophe increases with the stock of atmospheric carbon, this induces a target carbon tax of 22 US$/tCO2 with a linear hazard, and 57 $/tCO2 with a quadratic hazard, so that fossil-fuel use and the stock of atmospheric carbon are pushed down.

**Table 1:** After-disaster, naive and before-disaster target steady states (20% TFP shock; EIS = 0.5)

	After disaster	Naive solution	Constant hazard	Linear hazard	Quadratic hazard	Quadratic, EIS = 0.8
Capital stock (T $)	276	392	472	530	486	436
Consumption (T $)	41.3	58.6	59.4	59.6	59.2	58.9
Fossil fuel use (GtC/year)	7.3	10.4	11.0	9.7	7.7	7.7
Renewable use (million GBTU/year)	8.2	11.7	12.4	12.7	12.2	11.8
Carbon stock (GtC)	1218	1731	1838	1623	1281	1279
Precautionary return (%/year)	0	0	0.76	1.24	0.99	0.57
Carbon tax ($/tCO2)	0	0	0	22.4	56.9	51.0

The quadratic hazard has a higher target carbon tax because of the larger incentive to keep the carbon stock low. It follows that the target carbon stock is cut substantially below that for the linear hazard (1281 GtC instead of 1623 GtC). The quadratic hazard is thus decreased further below the linear hazard (3.8 instead of 6.5% per annum). Notice that the target precautionary return on capital is lower under the quadratic than under the linear hazard function (0.99 vs. 1.24% per annum). Hence, under the quadratic hazard function the target before-tip steady-state capital stock is only 486 trillion dollars whilst under the linear one it is 530 trillion dollars. The larger target capital stock will lead to a higher use of fossil fuel, and thus to a higher carbon tax, but the carbon tax remains substantially lower for the linear hazard than for the quadratic hazard (22.4 vs. 56.9 $/tCO2) because of the other effects.

With the quadratic hazard function the marginal hazard rate is slightly larger at the target level of the carbon stock than with the linear hazard (*H*_2_ʹ(1281) = 5.56 × 10^−5^ > *H*_1_ʹ(1623) = 5.04 × 10^−5^).^5^ This pushes up the carbon tax. Since the hazard rate itself is lower with the quadratic hazard, and thus the discount rate used to calculate the present value of the drop in value after a calamity is lower, the carbon tax is pushed up further.

**Fig. 2:**
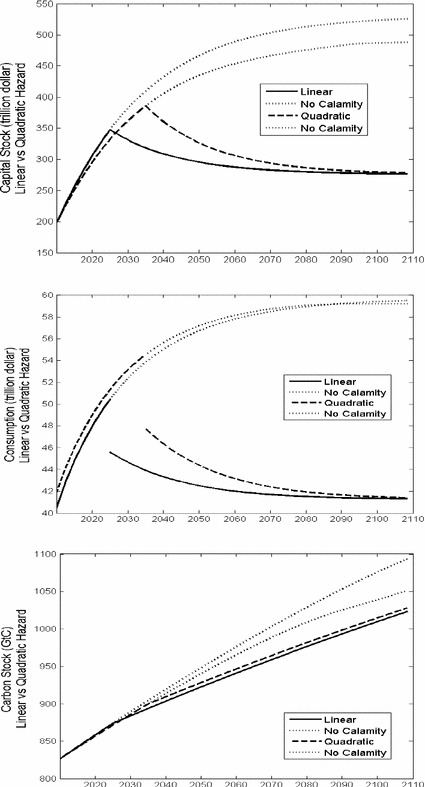
Rational outcomes with linear and quadratic hazard functions

The optimal time paths of capital, consumption and the accumulated carbon stock for the linear and quadratic hazard functions are plotted in Fig. 2. With global warming the hazard rates go up and the expected time of the regime shift is brought forward. This is especially the case for the linear hazard function and thus precautionary saving is higher to mitigate the effect of the shock. Hence, consumption is lower in the beginning and only catches up if the regime shift happens to occur late. With the quadratic hazard function, the hazard rate goes up more slowly in the relevant range. Moreover, the high carbon tax keeps the stock of atmospheric carbon down. The expected date of the regime shift occurs later than for the linear hazard rate and precautionary saving is not as high. If the linear hazard function applies, substantial precautionary saving and a moderate carbon tax are required. Despite the higher capital stock, and thus the higher use of fossil fuel, the carbon tax is moderate because the marginal hazard rate of an increased carbon stock is constant. If the quadratic hazard function applies, precautionary saving is lower but a higher carbon tax is required. The carbon tax is not so much needed to compensate for the consequences of the higher capital stock, but it is very much needed to keep the carbon stock down because the marginal hazard rate is increasing. For much more convex hazard functions, capital can be lower than in the naive outcome, so that precautionary saving is even turned upside down. For both hazard functions, the use of fossil fuel is less after than before the calamity as a result of a lower level of economic activity (even though the carbon is no longer taxed), and thus carbon accumulation occurs less rapidly.

In the last column of Table 1 we increase the elasticity of intertemporal substitution *EIS* from *σ* = 0.5 to *σ* = 0.8, or reduce the coefficients *CRIA* and *CRRA* from 2 to 1.25, for the quadratic hazard function. For the lower *CRIA*, we see substantial reductions in the precautionary return (from 0.99 to 0.57% per annum). This results in substantial drops in the target before-calamity capital stocks from 486 to 436 trillion dollars. As a result of the lower *CRRA*, we see only modest cuts in the carbon tax (from 56.9 to 51.0 $/tCO2). The target atmospheric carbon stock is curbed from 1281 to 1279 GtC. Less intergenerational inequality aversion curbs the target rate of consumption, but only by a very modest amount. In a companion paper (van der Ploeg and de Zeeuw 2018) we separate the coefficients of intergenerational inequality aversion (*CRIA*) and relative risk aversion (*CRRA*), using Duffie-Epstein preferences. In this way, the effects can be investigated of changing *CRIA* but keeping *CRRA* the same, and vice versa.

## Ramsey Growth with Climate Tipping: Gradual and Catastrophic Damages

In many integrated assessment models global mean temperature *Temp* decreases total factor productivity in a gradual way. Nordhaus (2008) uses $$ A = (1 + 0.00284\,Temp^{2} )^{ - 1} $$ in his DICE-2007 model, which implies damages of 1.7% of GDP at 2.5 °C. Temperature is often described by $$ Temp = \chi \ln (P/P_{PI} )/\ln (2) $$ where *χ* is the climate sensitivity (i.e., the temperature rise from doubling the carbon stock *P*) and *P*_*PI*_ = 596.4 GtC is the pre-industrial carbon stock, so that total factor productivity decreases in the carbon stock. Golosov et al. (2014) use *χ* = 3 (cf., IPPC 2007) and show that damages are well approximated by $$ A(P) = e^{{ - \xi (P - \bar{P})}} \bar{A}, $$ where *ξ* = 0.02379 US$/tC is the damage coefficient, $$ \bar{P} $$ the stock of carbon in 2010, and $$ \bar{A} $$ the total factor productivity in 2010. We can now derive and decompose the optimal carbon tax.

### **Proposition 1**

*The optimal carbon tax in the case of gradual and catastrophic damages becomes*:

13 $$ \begin{aligned} \tau (t) & = \underbrace {{\frac{\psi }{{U^{\prime } \left( {C^{B} (t)} \right)}}\int_{t}^{\infty } {e^{{ - \int_{t}^{s} {r^{U} (s^{\prime } )ds^{\prime } } }} H^{\prime}\left( {P(s)} \right)\left[ {V^{B} \left( {K(s),P(s)} \right) - V^{A} \left( {K(s),P(s)} \right)} \right]ds} }}_{{{\text{risk-averting}}\,{\text{part}}\,{\text{of}}\,{\text{the}}\,{\text{carbon}}\,{\text{tax}}}} \\ & \quad + \underbrace {{\xi \psi \int_{t}^{\infty } {e^{{ - \int_{t}^{s} {r^{Y} (s^{\prime } )ds^{\prime } } }} AF(s)ds} }}_{{{\text{conventional}}\,{\text{part}}\,{\text{of}}\,{\text{the}}\,{\text{carbon}}\,{\text{tax}}}} \\ & \quad + \underbrace {{\psi \int_{t}^{\infty } {e^{{ - \int_{t}^{s} {r^{Y} (s^{\prime } )ds^{\prime } } }} H\left( {P(s)} \right)\left[ {\frac{{ - V_{P}^{A} \left( {K(s),P(s)} \right)}}{{U^{\prime } \left( {C(s)} \right)}}} \right]ds} }}_{{{\text{raising-the-stakes}}\,{\text{part}}\,{\text{of}}\,{\text{the}}\,{\text{carbon}}\,{\text{tax}}}}. \\ \end{aligned} $$

### *Proof*

See “Appendix 4”.□

The first term in Eq. (13) represents the present value of the expected loss due to a future potential climate catastrophe. It is the same as Eq. (11), where we only had catastrophic damages. It curbs the carbon emissions and thus the risk of a climate calamity. Therefore, we call this the *risk*-*averting part* of the carbon tax. The second term is the usual present value of marginal climate damages, where the discount rate is equal to the rate of interest plus the decay rate of atmospheric carbon augmented with both the hazard rate of the catastrophe and the precautionary return.^6^ We call this the *conventional part* of the carbon tax. The third term arises because gradual damages are still relevant after the catastrophe. It reflects the expected present value of the after-calamity social cost of carbon, and thus yields the increase in after-calamity welfare from curbing the emissions by one unit. We call this the *raising*-*the*-*stakes part* of the carbon tax. We will now turn to what the extension with gradual damages implies for the numerical illustration with our calibrated model for the world economy.

### Decomposing the Carbon Tax

The first three columns of Table 2 give the after- and before-calamity steady states for both the linear and the quadratic hazard function. Comparing this with Table 1 shows the effects of adding marginal damages to the cost of global warming on the optimal carbon tax and macroeconomic outcomes. As a result of the marginal damages, after the calamity there is a conventional carbon tax of 11 $/tCO2 which curbs atmospheric carbon from 1502 to 1107 GtC. This slightly lowers capital and consumption after the calamity. Before the disaster strikes, there is a much bigger carbon tax for the linear hazard function (55 instead of 22 $/tCO2) than for the quadratic hazard function (71 instead of 57 $/tCO2), as compared to the situation without marginal climate damages. Adding the marginal climate damages mitigates the difference between the linear and quadratic hazard functions, because risk of tipping is not the only damage component anymore. The before-calamity carbon tax is in both cases dominated by the catastrophe components, especially the risk-averting component. The raising-the-stakes component is also high, and even higher than the after-calamity carbon tax. The aim of this carbon tax component is to keep the risk of after-calamity carbon costs low. Furthermore, as a consequence of introducing marginal climate damages, the carbon stock is much more reduced with the linear hazard function (from 1623 to 1287 GtC) than with the quadratic hazard function (from 1281 to 1161 GtC). Thus the differences between the effects of the two hazard functions become smaller.

**Table 2:** Target steady states with catastrophic and marginal damages

	Naive solution	20% shock in TFP			10% shock in TFP		
		After shock	Linear	Quadratic	After shock	Linear	Quadratic
Capital stock (T $)	378	271	492	465 (426)	323	431	421
Consumption (T $)	57.1	40.8	58.3	58.2 (58.0)	48.7	57.8	57.8
Carbon stock (GtC)	1502	1107	1287	1161 (1119)	1303	1425	1320
Temperature (°C)	4.00	2.68	3.33	2.88 (2.72)	3.38	3.77	3.44
Precautionary return (%/year)	0	0	1.10	0.90 (0.56)	0	0.57	0.49
Carbon tax ($/GtCO2)	15.4	11.0	54.8	71.2 (73.1)	13.2	29.8	41.5
*Marginal*	15.4	11.0	4.3	5.7 (8.5)	13.2	3.8	4.7
*Risk averting*	0	0	35.0	51.9 (54.8)	0	12.4	24.2
*Raising stakes*	0	0	15.4	13.7 (9.8)	0	13.7	12.5

In brackets are results for when the hazard rates are halved for each level of *P*

Internalising marginal damages as well as catastrophic damages curbs global warming and lessens the need for precautionary capital accumulation, especially for the linear hazard function (from 530 to 492 trillion dollars for the linear and from 486 to 465 trillion dollars for the quadratic hazard function). Hence, the precautionary return, largely driven by the hazard rate and carbon stock, drops by much more for the linear hazard (from 1.23 to 1.10% per annum) than for the quadratic hazard function (from 0.99 to 0.90% per annum). The convex hazard function has a lower marginal hazard rate at the target level of the carbon stock ($4.1 \times 10^{ - 5}$ for the quadratic and 5.0 × 10^−5^ for the linear hazard function), but the risk-averting component of the carbon tax is still higher as the hazard rate is lower. It is not so much higher now, since the hazard rates differ less between the linear and the quadratic hazard function.

The remaining columns of Table 2 show the consequences of a catastrophic drop of 10% in total factor productivity. Since there is more economic activity and more carbon emissions after the calamity than for the 20% disaster, the after-calamity carbon tax increases from 11 to 13 $/tCO2. Before the shock hits, both catastrophe components of the optimal carbon tax fall relative to the outcome with a 20% calamity, but the biggest falls occur for the risk-averting components (from 35 to 12 $/tCO2 for the linear and from 52 to 24 $/tCO2 for the quadratic hazard function). Although there is now more economic activity with a 10% shock after the calamity, the capital stock in the beginning is lower because there is less precautionary capital accumulation. The precautionary return drops more or less in line with the drop in total factor productivity so that less precautionary saving occurs before the catastrophe hits. Despite the lower initial capital stock after the calamity, the lower structural drop of only 10% in total factor productivity leads to bigger carbon stocks and more global warming.

In brackets we show how the results change if the quadratic hazard rates are halved for each level of the carbon stock. This curbs precautionary saving, but it increases the carbon tax by a small amount. The marginal hazard rates are lower but the hazard rates are lower as well and the last effect dominates so that the carbon tax increases.^7^

### Other Type of Shocks

#### Climate-Sensitivity Catastrophe

Instead of an economic catastrophe triggered by global warming we first consider here a climate catastrophe which induces an abrupt increase in the climate sensitivity *χ*.^8^ After the disaster, the carbon stock and the temperature increase and gradually erode productivity, which differs from the TFP catastrophe we have discussed so far where economic activity, fossil fuel use, emissions and eventually temperature drop after the tip (see “Appendix 5”). Table 3 shows the effects of a catastrophic increase in the climate sensitivity *χ* from 3 to 4 with a quadratic hazard.^9^ The after-calamity response requires a much higher carbon price than in the naive outcome (27 instead of 16 $/tCO2). Furthermore, before the calamity a substantial target carbon tax is needed (27 $/tCO2). This is not different from the after-calamity carbon tax, but there is a shift from marginal to raising-the-stakes damages. Once the calamity has struck, damages are higher and after-calamity welfare is lower, so that the raising-the-stakes component of the carbon tax is big. This in itself curbs carbon emissions and lessens the need for a correction of marginal damages. Moreover, before the calamity a small precautionary return is required and thus the target capital stock is a little bit higher than in the naive outcome (despite the higher carbon tax). The precautionary return is small because the shock to total factor productivity induced by the shock to climate sensitivity is not so big. Just as with the total factor productivity shocks, the carbon stock drops after the calamity. However, the after-calamity temperature is higher than for the productivity shock presented in Table 2.

**Table 3:** Target steady states for capital and carbon catastrophes

	Naive outcome	*CS* jumps from 3 to 4		20% jump in *P*	20% drop in *K*
		After calamity	Before calamity		
Capital stock (T $)	378	372	382	381	433
Consumption (T $)	57.1	56.3	57.3	57.1	57.6
Carbon stock (GtC)	1502	1374	1400	1490	1534
Temperature (°C)	4.00	4.82	3.69	3.96	4.09
Precautionary return (%/year)	0	0	0.05	0.03	0.57
Carbon tax ($/GtCO2)	15.4	26.7	26.5	16.9	18.5
*Marginal*	15.4	26.7	4.1	3.8	3.8
*Risk averting*	0	0	2.2	1.4	2.5
*Raising stakes*	0	0	20.2	11.7	12.2

#### Carbon-Stock Catastrophe

Table 3 also shows the effects of a hazard of a 20% increase in the carbon stock. There is little precautionary saving and the carbon tax is small (17 $/tCO2) because this is a *recoverable* catastrophe rather than a permanent regime shift. The carbon stock and global warming are cut somewhat below the naive outcome (1490 vs. 1502 GtC).

#### Capital-Stock Catastrophe

Finally, Table 3 shows the effects of a hazard of a 20% destruction of the capital stock. Now there is a sizeable precautionary return (0.57% per year) and substantial capital accumulation to prepare for sudden disaster (433 instead of 378 trillion dollars). Since this induces more fossil fuel use, there is despite a somewhat higher carbon tax (18.5 instead of 15.4 $/tCO2) *more* global warming than in the naive outcome (1534 GtC).

Before the calamity strikes, the risk-averting components of the carbon tax are tiny as damages caused by the catastrophe are temporary. Comparing with the naive outcome, the raising-the-stakes component takes over most of the marginal-damage component of the carbon tax, as we have also seen for the shock in the climate sensitivity. There is substantial accumulation of capital before the disaster strikes for an impending capital- stock disaster but unsurprisingly hardly any for an impending carbon-stock disaster.

### Adaptation Capital

The risk of a tipping point induces precautionary capital accumulation to be prepared when the shock hits. Alternatively, one can invest in specific adaptation capital *L* (e.g., seawalls, storm surge barriers, dune reinforcement and creation of marshlands as protection to sea level rises, crop relocation, diversifying tourist attractions, adjusting rail and roads to cope with warming and drainage).^10^ Adaptation capital *L* reduces the shock, so *π* = *Π*(*L*) with Πʹ(*L*) < 0. Suppose that both types of capital are *ex ante* perfect substitutes and have the same depreciation rate, *δ*. We assume a “putty-clay” technology: from the tipping point onwards, *L* is constant as there is no need to cover for depreciation and *L* cannot be turned *ex post* into productive capital. We ignore marginal damages, so after-calamity does not depend on the carbon stock. The right-hand side of the Hamilton–Jacobi-Bellman equation becomes

14 $$ \varOmega \equiv \mathop {\text{Max}}\limits_{C,L} \left[ {U(C) + V_{K}^{B} (K,P)\left\{ {Y^{B} (K - L,P) - C} \right\} - H(P)\left\{ {V^{B} (K,P) - V^{A} \left( {K - L,\varPi (L)} \right)} \right\}} \right], $$

with the condition for the optimal stock of adaptation capital,^11^

15 $$ Y_{K - L}^{B} (K - L,P) = \frac{{H(P)\left[ {V_{\pi }^{A} \left( {K - L,\varPi (L)} \right)\varPi^{\prime } (L) - V_{K - L}^{A} \left( {K - L,\varPi (L)} \right)} \right]}}{{V_{K}^{B} (K,P)}}. $$

We define $\hat{V}_{L}^{A} (K,L) \equiv V_{\pi }^{A} \left( {K - L,\varPi (L)} \right)\varPi^{\prime } (L) - V_{K - L}^{A} \left( {K - L,\varPi (L)} \right) > 0$ and suppose $\hat{V}_{L}^{A} > 0,$ so that the total marginal return on adaptation capital is decreasing in *L* and increasing in *K*, i.e. $V_{LL}^{A} < 0$ and $V_{LK}^{A} > 0.$ The share of adaptation capital *L* is zero if the net effect of a lower shock *Π*(*L*) and a lower productive capital *K*–*L* after the event cannot be positive. Otherwise, (15) requires that the expected marginal return on adaptation capital in final goods units must be equal to the marginal productivity of capital used in production. The optimal amount of adaptation capital then increases in both capital and carbon stocks:

16 $$ L = L(P,K){\text{ with }}L_{P} > 0{\text{ and }}L_{K} > 0. $$

If the hazard rate is invariant to global warming, (16) becomes $L = L(h,K)$ with *L*_*h*_ > 0 and *L*_*K*_ > 0. A higher hazard rate *h* induces precautionary capital accumulation. Here we see that a bigger risk of catastrophe leads to more adaptation capital *L*. In general, the hazard rate increases with global warming in which case the optimal level of adaptation capital and the aggregate capital stock increase with the carbon stock.

## Conclusion

Climate change will probably manifest itself in the future as a regime shift in the climate system resulting from a climate tipping point (Lenton and Ciscar 2013). This means that the risk of such a shock to the economy becomes an important driver of the social cost of carbon. It has also been argued that spending money now to slow down global warming should be conceptualised primarily as an issue about how much insurance to buy to offset the small chance of a ruinous catastrophe (Weitzman 2007). We have taken up this challenge by analysing the optimal reactions to a tipping point in a Ramsey growth model which becomes more imminent with global warming.

The most striking result in this literature is that both a carbon tax and a precautionary adjustment to capital are needed. A reduction in fossil fuel use is required to curb the risk of a catastrophe and adjustment to capital is required to smooth consumption over time. In combination with the standard Pigouvian carbon tax, this carbon tax also has a third component because the regime shift increases the marginal damage after the calamity. Higher elasticity of intertemporal substitution (or lower risk aversion c.q. lower intergenerational inequality aversion) decreases both the carbon tax and the precautionary adjustments to capital.

Regime shifts are characterised in our analysis as structural shocks either to total factor productivity or to climate sensitivity. We show that the effects on optimal policy are much larger than in case of shocks to the capital stock or the stock of atmospheric carbon. The last two cases only set back development temporarily and do not affect the steady state of the economy, so that there is less need for the optimal policy to curb the risk of tipping.

A constant marginal hazard rate requires a balanced package of a carbon tax and a precautionary adjustment to saving. However, for an increasing marginal hazard rate a higher carbon tax is needed, so that the stock of atmospheric carbon is kept down and precautionary adjustments to capital can be more modest.

Our results highlight the importance of a carbon tax to curb the risk of a catastrophe, and precautionary adjustments to capital to be prepared when the disaster strikes. Our illustrative calculations indicate that especially the risk-averting part of the carbon tax is substantial as compared to the carbon taxes based on only marginal damages (e.g., Nordhaus 2008; Golosov et al. 2014).

Our conclusion is that destruction of non-recoverable factors of production by climate catastrophes requires urgent action both to mitigate the risks of such actions occurring and to be better prepared for when they occur. Our illustrative calculations suggest that conventional marginal global warming damages necessitate a global carbon tax of 15 $/tCO2.^12^ However, an impending negative shock of 20% to total factor productivity with an expected arrival time in 40 years, falling to 15 years when the carbon stock doubles, boosts this figure to 55 or 71 $/tCO2 for a linear or quadratic hazard function, respectively. Furthermore, a precautionary return of about 1% per annum induces an additional capital accumulation of 30 and 23%, respectively. A negative shock of 10% requires a global carbon tax of 30–40 $/tCO2 and also halves the precautionary returns. Half the hazard rates for any given carbon stock hardly changes the required carbon tax, but diminishes the precautionary return considerably to 0.16% per annum. Catastrophic shocks to recoverable factors of production such as the capital stock are only temporary and thus require much less action.

Catastrophes might resonate more with policy makers to convince them of the need to implement ambitious climate policy. However, as argued by Lenton and Ciscar (2013), more research with climate scientists is needed to improve information on the different types of productivity, capital-stock, carbon-stock and climate-sensitivity catastrophes that can occur, and the different hazard and marginal hazard rates of such disasters. We also need to be precise about how long it will take before the impact of the catastrophe is fully felt (Lontzek et al. 2015). In a companion paper (van der Ploeg and de Zeeuw 2018), we introduce a delay for the catastrophe to have its full impact. Our main result is that for a sufficiently large impact delay, the precautionary return is negative, so that less saving before the tip occurs and consumption jumps up at the tipping point.

Future research is thus needed on the nature of catastrophes, on their hazard rates and their dependence on global warming, on the time it takes to have their effect, and how these insights affect the optimal policy prescriptions for dealing with climate tipping points. More thinking is also required on what kinds of abatement or mitigation investments can be made today that yield a positive return in the distant future at the time the catastrophe strikes. In other words, one needs projects that yield a negative “climate beta”. These occur if a bad climate shock is associated to higher temperature and lower economic growth, in which case there is an insurance motive to push up the optimal price of carbon (e.g., Sandsmark and Vennemo 2007; Daniel et al. 2016). However, in most integrated assessment models, damages and GDP are tied to each other and thus the “climate beta” is positive and close to one, in which case it is easier to deal with future climate shocks and climate policy can be relaxed (e.g., Dietz et al. 2017). Another difficulty occurs from a finance perspective due to the problem of “maturity mismatch”: climate change occurs over the course of the next few centuries whereas returns on investments in physical capital might have a horizon of more like half a century. Some forms of adaptation capital might however give a return when the catastrophe hits and typically last for much longer than other types of capital.

## Figures and Tables

**Fig. 3:**
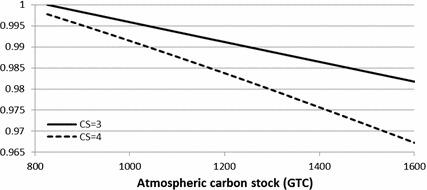
Effects of climate sensitivity on global warming damages

## Appendix 1: After the Climate Shift

First note that the optimal choice of fossil fuel *E* and renewable energy *R* is made at each point in time, without affecting the dynamics of the problem. Therefore, we can simplify the problem by defining the maximum level of output *Y net* of input costs and capital depreciation:

A1 $$ Y(K,\pi ) \equiv \mathop {\text{Max}}\limits_{E,R} \;\left[ {(1 - \pi )AF(K,E,R) - dE - cR - \delta K} \right]. $$

For brevity, we suppress the arguments *A, c* and *d* in the maximal net output function, but note that energy use is given by $$ E^{A} = - Y_{d} > 0 $$ and $$ R^{A} = - Y_{c} > 0, $$ where the after-calamity values are denoted with superscript *A*. Energy use increases with the capital stock *K* and decreases in the size of the disaster *π* and the own price, so that $$ Y_{iK} < 0 $$ and $$ Y_{i\pi } > 0,\;i = d,c. $$

The Hamilton–Jacobi–Bellman (HJB) equation in the value function *V*^*A*^ is

A2 $$ \rho V^{A} (K,\pi ) = \mathop {\text{Max}}\limits_{C} \;\left[ {U(C) + V_{K}^{A} (K,\pi )\left\{ {Y(K,\pi ) - C} \right\}} \right]. $$

Optimality requires $U'(C^{A} ) = V_{K}^{A} (K,\pi ),$ which gives the policy rule $C^{A} (K,\pi ).$

Differentiating (A2) with respect to *K*, using this optimality condition and a *CRRA* utility function *U*, yields a differential equation for consumption after the regime shift *C*^*A*^ as a function of *K*:

A3 $$ \left[ {Y(K,\pi ) - C^{A} (K,\pi )} \right]C_{K}^{A} (K,\pi ) = \sigma \left[ {Y_{K} (K,\pi ) - \rho } \right]C^{A} (K,\pi ), $$

where $\sigma \equiv - U^{\prime } /CU^{{\prime \prime }} > 0$ is the elasticity of intertemporal substitution. Both relative risk aversion and relative intergenerational inequality aversion are equal 1/*σ*. Equation (A3) can be written as a saddle-point system of differential equations in *C* and *K* as functions of time corresponding to the Keynes–Ramsey rule and the capital dynamics:

A4 $$ \begin{aligned} \dot{C}^{A} (t) & = \sigma \left[ {Y_{K} \left( {K(t),\pi } \right) - \rho } \right]C^{A} (t), \\ \dot{K}(t) & = Y\left( {K(t),\pi } \right) - C^{A} (t),\quad K(T) = K_{T} . \\ \end{aligned} $$

The steady-state capital stock $K^{A*} (\pi )$ follows from the modified golden rule of capital accumulation $Y_{K} (K^{A*} ,\pi ) = \rho ,$ and is low if the disaster is large and the discount rate is high. As far as the transient phase is concerned, the capital stock is predetermined at time *T*, but consumption jumps up or jumps down to place the economy on the stable manifold, $C^{A} (T + ) = C^{A} \left( {K(T + ),\pi } \right)$. It depends on the parameter values whether the consumption jumps up or jumps down. For reasonable parameter values, as in the calibration in this paper, consumption jumps down. Rearranging (A2) gives the value function:

A5 $$ V^{A} (K,\pi ) = \frac{{U\left( {C^{A} (K,\pi )} \right) + U'\left( {C^{A} (K,\pi )} \right)\left[ {Y(K,\pi ) - C^{A} (K,\pi )} \right]}}{\rho }. $$

The optimal path along the stable manifold is $C^{A} (t) = C^{A} \left( {K(t),\pi } \right),\;t \ge T.$ In the sequel of the paper we use the log-linear approximation

A6 $$ C^{A} = C^{A} (K,\pi ) \cong Y\left( {K^{{A^{*} }} (\pi ),\pi } \right)\left( {\frac{K}{{K^{{A^{*} }} (\pi )}}} \right)^{\phi } ,\quad \phi \equiv z\frac{{K^{{A^{*} }} (\pi )}}{{Y\left( {K^{{A^{*} }} (\pi ),\pi } \right)}} > 0, $$

where the direction of the stable manifold, $z \equiv C_{K}^{A} (K^{{A^{*} }} ,\pi )$, is derived from (A3) with l’Hôpital’s rule:

A7 $$ C_{K}^{A} (K^{{A^{*} }} ,\pi ) = \mathop {\lim }\limits_{{K \to K^{{A^{*} }} }} \frac{{\sigma \left[ {Y_{K} (K,\pi ) - \rho } \right]C_{K}^{A} (K,\pi ) + \sigma Y_{KK} (K,\pi )C^{A} (K,\pi )}}{{Y_{K} (K,\pi ) - C_{K}^{A} (K,\pi )}}. $$

This yields the quadratic $z^{2} - \rho z + \sigma Y_{KK} (K^{{A^{*} }} ,\pi )C^{{A^{*} }} = 0.$ The positive solution yields the slope $z = \frac{\rho }{2} + \frac{1}{2}\sqrt {\rho^{2} - 4\sigma Y_{KK} (K^{{A^{*} }} ,\pi )C^{{A^{*} }} } > \rho > 0$ of the stable manifold in the steady state. Although we could solve the after-calamity problem by using value function iteration to solve the HJB equation, the approximation (A6) is accurate and convenient.

## Appendix 2: Before the Climate Shift

Again we define $Y^{B} (.)$ as the maximum level of output *net* of total input costs and capital depreciation:

A.8 $$ Y^{B} (K,\tau ) \equiv \mathop {\text{Max}}\limits_{E,R} \;\left[ {AF(K,E,R) - (d + \tau )E - cR - \delta K} \right], $$

so that the efficiency conditions for energy use are $AF_{E} = d + \tau$ and *AF*_*R*_ = *c*. The cost of fossil fuel is thus augmented with the social cost of carbon. If carbon is taxed at the social cost of carbon, this condition for the social optimum is replicated in the market.

Differentiating (6) with respect to *K* and *P*, using (7), gives the Pontryagin conditions:

A.9 $$ \begin{aligned} - \dot{V}_{K}^{B} & = \left[ {Y_{K}^{B} (K,\tau ) - \rho - H(P)} \right]V_{K}^{B} + H(P)V_{K}^{A} (K), \\ \dot{V}_{P}^{B} & = \left[ {\rho + \gamma + H(P)} \right]V_{P}^{B} + H^{\prime}(P)\left[ {V^{B} (K,P) - V^{A} (K)} \right]. \\ \end{aligned} $$

Using (8) and (A.9) yields the differential Eq. (9) for the price of carbon *τ*. Using (A.9), (7), and a *CRRA* utility function *U* with elasticity of intertemporal substitution *σ*, yields the modified Keynes–Ramsey rule (10).

The accumulation of capital and the stock of carbon can be written as

A.10 $$ \begin{aligned} & \dot{K} = Y^{B} (K,\tau ) - \tau Y_{\tau }^{B} (K,\tau ) - C^{B} ,\quad K(0) = K_{0} , \\ & \dot{P} = - \psi Y_{\tau }^{B} (K,\tau ) - \gamma P,\quad P(0) = P_{0} . \\ \end{aligned} $$

The capital dynamics include lump-sum rebates of carbon taxes in a market economy.

The steady state of the 4-dimensional Hamiltonian system (9), (10) and (A.10) is indicated with an asterisk and follows from the modified golden rule of capital accumulation $Y_{K}^{B} (K^{B*} ,\tau^{B*} ) = \rho - \theta^{B*}$, leading to the precautionary return on capital adjustments $\theta^{B*} = H(P^{B*} )\left[ {\frac{{V_{K}^{A} (K^{B*} )}}{{U'(C^{B*} )}} - 1} \right],$ the carbon tax

$$ \tau^{B*} = \frac{{\psi H^{\prime}(P^{B*} )\left[ {V^{B} (K^{B*} ,P^{B*} ) - V^{A} (K^{B*} )} \right]}}{{\left[ {\rho + \gamma + H(P^{B*} )} \right]U'(C^{B*} )}} = \frac{{\psi H^{\prime}(P^{B*} )\left[ {U(C^{B*} ) - \rho V^{A} (K^{B*} )} \right]}}{{\left[ {\rho + H(P^{B*} )} \right]\left[ {\rho + \gamma + H(P^{B*} )} \right]U'(C^{B*} )}}, $$

the rate of consumption $C^{B*} = Y^{B} (K^{B*} ,\tau^{B*} ) - \tau^{B*} Y_{\tau }^{B} (K^{{B^{*} }} ,\tau^{B*} )$ and the carbon stock $P^{B*} = - \psi Y_{\tau }^{B} (K^{B*} ,\tau^{B*} )/\gamma$. This gives a *target* steady state as after the regime shift the system moves to the after-calamity steady state $K^{A*} .$

## Appendix 3: Calibration

We use a utility function *U* with constant elasticity of intertemporal substitution *σ* = 0.5 (and 0.8 in a sensitivity test) and a pure rate of time preference *ρ* = 0.014. Our parameters and initial values are calibrated to figures for the world economy for the year 2010. Data sources are the BP Statistical Review and the World Bank Development Indicators. Output is *AF* before and (1–*π*)*AF* after the disaster, the Cobb–Douglas production function is $$ F(K,E,R) = K^{\alpha } (E^{\omega } R^{(1 - \omega )} )^{\beta } $$ with share of capital in value added *α* = 0.3, share of fossil fuel in energy *ω* = 0.9614 and share of energy in value added *β* = 0.0651. The share of fossil fuel and labour in value added are thus *βω* = 0.0626 and 1 – *α* – *β* = 0.6349. We use a depreciation rate for manmade capital of *δ* = 0.05. Total factor productivity is set to *A* = 11.9762, so that initial world GDP equals *Y*_0_ = 63 trillion US $. The corresponding initial value of the aggregate capital stock is *K*_0_ = 200 trillion US $, initial fossil use is *E*_0_ = 468.3 million GBTU or 8.3 GtC, and initial renewable energy use is *R*_0_ = 9.4 million GBTU. The calibrated production share parameters are compatible with a cost of fossil fuel of *d* = 9 US $/million BTU or 504 US $/tC and a cost of renewable energy of *c* = 18 US $/million BTU. For the carbon cycle we have an initial stock of carbon of *P*_0_ = 826 GtC or 338 ppm by volume CO2, a rate of decay *γ* = 0.003, a fraction of carbon staying up in the atmosphere of *ψ* = 0.5, and an equilibrium climate sensitivity *χ* = 3 (or 4 in a sensitivity run). The catastrophic shock to total factor productivity is *π* = 0.2 (or 0.1 in a sensitivity run).

## Appendix 4: Proof of Proposition 1

$V^{A} (K,P,\pi )$ requires $\tau^{A} (K,P,\pi ) \equiv - \psi V_{P}^{A} (K,P,\pi )/U'(C) > 0$ to internalise gradual damages. After-calamity consumption *C*^*A*^(*K*, *P*, *π*) yields

A.11 $$ V^{A} (K,P,\pi ) = U\left( {C^{A} (K,P,\pi )} \right)/\rho + U^{\prime } \left( {C^{A} (K,P,\pi )} \right)\left[ {Y^{A} (K,P,\pi ) - C^{A} (K,P,\pi ) + \gamma \tau^{A} (K,P,\pi )P/\psi } \right]/\rho . $$

As a consequence, the second parts of (A.9) and (9) become:

A.12 $$ \dot{V}_{P}^{B} = \left[ {\rho + \gamma + H(P)} \right]V_{P}^{B} + H^{\prime}(P)(V^{B} - V^{A} ) - A'FV_{K}^{B} - H(P)V_{P}^{A} , $$

A.13 $$ \dot{\tau } = \left[ {Y_{K}^{B} (K,\tau ) + \gamma + H(P) + \theta } \right]\tau - \frac{\psi }{{U'(C^{B} )}}\left[ {H^{\prime}(P)(V^{B} - V^{A} ) - A'FV_{K}^{B} - H(P)V_{P}^{A} } \right]. $$

Integrating (A.13), we get (13). The target before-calamity steady-state carbon tax is

$$ \tau^{B*} = \tau_{\text{conventional}}^{B*} + \tau_{\text{risk-averting}}^{B*} + \tau_{\text{raising-the-stakes}}^{B*} ,\quad {\text{where}}\quad \tau_{\text{conventional}}^{B*} \equiv \frac{{\xi \psi A(P^{B*} )F(K^{B*} ,\tau^{B*} )}}{{\rho + \gamma + H(P^{B*} )}}, $$

$$ \tau_{\text{risk-averting}}^{B*} \equiv \frac{{\psi H^{\prime}(P^{B*} )\left[ {U(C^{B*} ) - \rho V^{A} (K^{B*} ,P^{B*} )} \right]}}{{\left[ {\rho + \gamma + H(P^{B*} )} \right]\left[ {\rho + H(P^{B*} )} \right]U'(C^{B*} )}}, $$

$$ \tau_{\text{raising-the-stakes}}^{B*} \equiv \frac{{H(P^{B*} )\tau^{A} (K^{B*} ,P^{B*} )U'\left( {C^{A} (K^{B*} ,P^{B*} )} \right)}}{{\left[ {\rho + \gamma + H(P^{B*} )} \right]U'(C^{B*} )}}, $$

and *A*(*P*)*F*(*K*,*τ*) is the maximum level of gross output.

## Appendix 5: Carbon Catastrophes

Our damages and *χ* = 3 imply $A\left( {Temp} \right) = \bar{A}\exp \left[ { - \xi \left( {2^{Temp/3} P_{PI} - \bar{P}} \right)} \right].$ A catastrophic increase in the climate sensitivity (*CS*) to *χ* > 4 raises the temperature response and thus increases after-calamity damages as can be seen from substituting the temperature response: $A(P) = \bar{A}\exp \left[ { - \xi \left( {\left( {P/P_{PI} } \right)^{{\frac{\chi }{3}}} P_{PI} - \bar{P}} \right)} \right].$ The catastrophic rise in *χ* pushes up damages ($A^{\prime } (P) = - \xi (\chi /3)\left( {P/P_{PI} } \right)^{(\chi - 3)/3} A(P) < - \xi A(P) < 0$).

Figure 3 indicates that a bigger climate sensitivity of 4 (the dashed line) than our benchmark of 3 (the solid line) leads, for a given carbon stock, to bigger damages, with the hazard function unaltered. A sudden increase in climate sensitivity thus leads to a tougher challenge. Doubling the initial carbon stock induces a 2.0% drop in total factor productivity if climate sensitivity is 3 and a 3.4% drop if the climate sensitivity is 4.

## Footnotes

1 The ice-albedo effect acts more quickly over oceans than land as sea ice melts faster than continental ice sheets. Also, the demise of rain forests curbs transpiration as plants have lower reflectivity than soil.

2 About a fifth of carbon emissions remain in the atmosphere for thousands of years or forever, but in contrast to Golosov et al. (2014) we suppose that all of it decays eventually. We also ignore temperature lags, but see Gerlagh and Liski (2018) for that. Our calibration of (3) below is close to that of the seminal contribution of Nordhaus (1991), who uses *ψ* = 0.5 and *γ* = 0.005. We set *ψ* = 0.5 and *γ* = 0.003.

3 For simplicity, we suppress the dependence of the value functions on *π* from now on.

4 Calibration details are in “Appendix 3”. In the simulations we set *θ* and *τ* to their steady-state levels.

5 With a 30% shock to total factor productivity the marginal hazard for the quadratic hazard function is smaller. Still, the optimal target carbon tax is bigger due to the lower hazard rate and thus lower discount rate used to discount expected climate damages.

6 With logarithmic utility, Cobb–Douglas production and 100% depreciation, this part of the SCC in a discrete-time Ramsey model is proportional to GDP, $\tau = \psi \xi AF/(\rho + \gamma )$ (Golosov et al. 2014).

7 With a linear hazard function (not reported in Table 2) there is still substantial precautionary capital accumulation after halving hazard rates (437 T$). The carbon tax is a little higher for the linear (77.2 $/tCO2) than for the quadratic hazard function, mainly due to the risk-averting component as the marginal hazard for the linear hazard function is higher than for the quadratic function (2.52 × 10^−5^ > 1.78 × 10^−5^). The interplay between the marginal hazard and the hazard rate itself can work out in different ways, but it is clear that a big carbon tax is needed to curb the risk of catastrophe.

8 An alternative is to have abrupt positive feedback by changing the system dynamics of the carbon cycle as discussed in Lemoine and Traeger (2014) and van der Ploeg (2014).

9 Responses with a linear hazard function hardly differ from those with a quadratic hazard function.

10 A third option is to invest in mitigation capital to curb the hazard of climate change (e.g., institutions, norms and networks that facilitate the implementation of optimal climate policy, but also CCS).

11 Condition (15) follows from $\varOmega_{L} = - V_{K}^{B} Y_{K - L}^{B} + H(P)\hat{V}_{L}^{A} = 0.$ The second-order condition is $\varOmega_{LL} = V_{K}^{B} Y_{K - L,K - L}^{B} + H(P)\hat{V}_{LL}^{A} < 0.$ Since $\varOmega_{LP} = H^{\prime}(P)\hat{V}_{L}^{A} > 0,$ we have $L_{P} = - \varOmega_{LP} /\varOmega_{LL} > 0.$ Since $\varOmega_{LK} = - V_{K}^{B} Y_{K - L,K - L}^{B} + H(P)\hat{V}_{LK}^{A} - V_{KK}^{B} Y_{K - L}^{B} > 0,$ so $L_{K} = - \varOmega_{LK} /\varOmega_{LL} > 0.$

12 This is not too different from the latest ballpark measure of 12 $/tCO2 (including uncertainty, equity weighting and risk aversion) given by Nordhaus (2014).
